# Supplementary material for: Prostate Cancer: A Journey Through Its History and Recent Developments
Source: Cancers (Basel). 2025 Jan 9;17(2):194. doi: 10.3390/cancers17020194 (PMC11763992; doi:10.3390/cancers17020194)
Supplement: Supplementary file 1 [file cancers-17-00194-s001.zip › Table S1 and Table S2.pdf]

Year	Compound	Characteristics of study	Types of treatment	mechanism	Outcomes	References
2004	Docetaxel<br><br>Mitoxantrone (control)	674 patients:<br>338 (docetaxel every 3 weeks)<br>336 (mitoxantrone every 3 weeks)	Docetaxel (first line of chemotherapy)<br><br>Mitoxantrone (second line of chemotherapy)	Tubulin inhibition<br><br>Blocking DNA synthesis and repair	Median survival than treatment with mitoxantrone and prednisone (17.5 months vs. 15.6 months)<br>Hazard ratios (0.80 and 0.76, respectively)	(89)
2004	Docetaxel<br><br>Mitoxantrone (control)	1006 patients:<br>335 (docetaxel every 3 weeks)<br>337 (mitoxantrone every 3 weeks)	Docetaxel (first line of chemotherapy)<br><br>Mitoxantrone (second line of chemotherapy)		Median survival, 18.9 vs. 16.5 months;<br>Hazard ratio [HR] = 0.76	(103)
2006, 2010	Sipuleucel-T	In 2004,127 patients<br>82 :sipuleucel-T or<br>45 placebo	Immunotherapy	Vaccine	25.9 months in the sipuleucel-T group vs. 21.4 months in the placebo group	(114,115,161)
2010	Cabazitaxel<br><br>Mitoxantrone	755 men were allocated to treatment groups (377 mitoxantrone, 378 cabazitaxel)	Chemotherapy	Tubulin inhibition	median survival:<br>15·1 months in the cabazitaxel group and 12·7 months in the mitoxantrone group	(92)
2013	Radium-223	921 patients:<br>614 patients take radium 223 and 307 placebo	Radiotherapy	Targeting bone metastasis	Median survival 14.0 months vs. 11.2 months	(113)

2015	Abiraterone acetate<br><br>Placebo (control)	1088 patients:<br>546 (abiraterone acetate 1 g daily)<br>542 placebo controls	Hormonotherapy	Reducing androgen production	Median overall survival was significantly longer in the abiraterone acetate group than in the placebo group (34·7 months vs 30·3 months<br>hazard ratio 0·81	(162)
2017,2020	Enzalutamide<br>Placebo (control)	1717 patients:<br>872 take Enzalutamide<br>845 placebo controls	Hormonotherapy	Reducing androgen production	Median OS of enzalutamide vs placebo (36 vs 31 months)	(163,164)
2019	Cabazitaxel<br><br>Abiraterone or enzalutamide based on first line	255 patients:<br>129 patients in the cabazitaxel group<br>26 patients in the group that received an androgen-signaling-targeted inhibitor	Chemotherapy + Hormotherapy		The median overall survival was 13.6 months with cabazitaxel and 11.0 months with the androgen-signaling-targeted inhibitor	(165)

Table S1 : characteristics of main and second line treatment of mCRPC.

Year	Compound	Characteristics of study	Types of treatment	Outcomes	References
2012	Abiraterone Acetate after Docetaxel (phase III)	1195 patients, 797: abiraterone acetate plus prednisone (abiraterone group) and 398: placebo plus prednisone (placebo group)	Hormonotherapy + chemotherapy	The abiraterone group had a longer median overall survival (15·8 months versus 11·2 months [10·4-13·1]; hazard ratio [HR] 0·74; p<0·0001) in comparison to the placebo group.	(110)
2012	Enzalutamide after Docetaxel (phaseIII)	1199 men with castration-resistant prostate cancer after chemotherapy<br>800 patients: (Enzalutamide group), 399 patients (placebo group)	Hormonotherapy + chemotherapy	the enzalutamide group had a median of 18.4 months (95% CI: 17.3 to not yet reached) while the placebo group had a median of 13.6 months (95% CI: 11.3 to 15.8) (hazard ratio for death in the enzalutamide group, 0.63; 95% CI: 0.53 to 0.75; P<0.001).	(166)
2015	Abiraterone Acetate Prior to Docetaxel<br>(phase III)	1088 patients with chemotherapy-naive prostate cancer<br>546 patients (abiraterone acetate group)<br>542 (placebo group)	Hormonotherapy + chemotherapy	OS improved at a median of 49.2 months (34.7 vs. 30.3 months,	(162)

2015	Enzalutamide Prior to Docetaxel(phaseIII)	1717 patients, 872 (Enzalutamide group)<br>845 (Placebo group)	Hormonotherapy + chemotherapy	Enzalutamide was associated with a higher OS compared to placebo (32.4 months versus 25.1 months; hazard ratio (HR) = 0.61 (95% CI 0.47-0.79); P = 0.0001), rPFS was higher with enzalutamide than with placebo (not reached (95% CI 12.3-not yet reached) versus 3.7 months (95% CI 3.6-5.3); HR = 0.17 (95% CI 0.12-0.24); P < 0.0001).	(167)
2020	Rucaparib	115 patients (BRCA mutated)	PARPi	ORRs per independent radiology review and investigator assessment were 43.5% (95% CI, 31.0% to 56.7%; 27 of 62 patients) and 50.8% (95% CI, 38.1% to 63.4%; 33 of 65 patients), respectively.<br>PSA response rate 54.8%	(168)
2020	Pembrolizumab	258 patients:<br>133 group I (PDL-1 positive)	Immunotherapy	OS: 9.5 (group I) vs 7.9 (group	(121,122)

		66 group II (PDL1 negative)<br>59 group III (Bone metastasis)		II) vs 14.1 (group III)	
2017, 2020	Olaparib	2792 Patients	PARPi	In an interim analysis , the median overall survival 17.5 months in the Olaparib group and 14.3months in the control group	(118)
2021	Ipatasertib	1101 patients :<br>554 patients (Abiraterone + placebo<br>547 (abiraterone + ipatasertib)	PI3K/AKT/ mTOR inhibitors	PFS:<br>18.5 vs 16.5 months	(119)
2021	Talozoparib	128 patients (HRR mutations)	PARPi	objective response (OS) rate 29·8% (31 of 104 patients; 95% CI 21·2-39·6)	(169)
2021	Capivasertib	150 patients:<br>75 Capivasertib<br>75 placebo	PI3K/AKT/ mTOR inhibitors	OS: 31.1 vs 20.2 months<br>PFS: 7.03 vs 6.7 months	(170)
2021	Lu-PSMA-617	831 patients:<br>551 (Lu-PSMA-617)<br>280 controls	Theragnostic	OS: 15.3 vs 11.3 months<br>PFS: 8.7 vs 3.4 months	(123)
2023	Niraparib	223 patients:<br>142 with<br>HRR mutations<br>81 with other HRR mutations	PARPi	The median survival 10.1 months 5h (HRR mutation) vs 8.6 months (other mutation)	(171)
2023	Talazoparib + Enzalutamide	805 patients<br>402 talazoparib + enzalutamide<br>403 placebo with enzalutamide	Hormonotherapy + PARPi	median rPFS was not reached (27·5 months-not reached) for	(124)

				talazoparib plus enzalutamide<br>21·9 months (16·6-25·1) for placebo plus enzalutamide	
2023	Niraparib + Abiraterone	423 patients<br>212 niraparib with abiraterone<br>211 placebo with abiraterone	Hormonotherapy + PARPi	rPFS in niraparib + abiraterone group compared with the placebo + Abiraterone group (16.5 *v* 13.7 months)	(125)
2023	Olaparib with abiraterone	1103 Patients :<br>399 patients olaparib plus abiraterone 397 patients placebo plus abiraterone	Hormonotherapy + PARPi	Median overall survival 42·1 months with Olaparib plus abiraterone and 34·7 months with placebo plus abiraterone	(126)

Table S2: Characteristics of other lines treatment of mCRPC
