# Supplementary material for: Prostate Cancer: A Journey Through Its History and Recent Developments
Source: Cancers (Basel). 2025 Jan 9;17(2):194. doi: 10.3390/cancers17020194 (PMC11763992; doi:10.3390/cancers17020194)
Supplement: Supplementary file 1 [file cancers-17-00194-s001.zip › Table S3.pdf]

Trial Name	Phase	Status	Agents Tested	Notable Results/Outcomes	References
TRANSFORM	N/A	Active	Fast MRI scans, genetic testing, PSA blood testing	Largest PC screening trial in 20 years, comparing multiple screening options	[151]
ENACT	2	Inactive	Enzalutamide monotherapy	46% reduced risk of PC progression vs. active surveillance	[152]
XmAb20717 (Vudalimab)	2	Active	Vudalimab (XmAb20717)	Evaluating efficacy in advanced gynecologic and genitourinary malignancies	[153]
Olaparib + LHRH agonist	2	Active	Olaparib, LHRH agonist	Neoadjuvant therapy for high-risk localized PC	[154]

**Trial Name**	**Phase**	**Status**	**Agents Tested**	**Notable Results/Outcomes**	**References**
Xaluritamig (AMG509)	1	Active	STEAP1 × CD3 bispecific antibody	First-in-human study for metastatic castration-resistant PC	[155]

Table S3. Overview of both active and inactive clinical trials in PC in 2023-2024.
